# Karachi Cancer Registry (KCR): Age-Standardized Incidence Rate by Age-Group and Gender in a Mega City of Pakistan

**DOI:** 10.31557/APJCP.2020.21.11.3251

**Published:** 2020-11

**Authors:** Shahid Pervez, Adnan A Jabbar, Ghulam Haider, Shamvil Ashraf, Muhammad Asif Qureshi, Fouzia Lateef, Imtiaz Bashir, Manzoor Zaidi, Muhammad Khurshid, Mohammed Saeed Quraishy, Tariq Siddiqi, Uzma Rizwan, Muhammad Arif Nadeem Saqib, Muhammad A Memon, Ejaz Alam, Huma Qureshi

**Affiliations:** 1 *Department of Pathology & Laboratory Medicine, Aga Khan University Hospital, Karachi, Pakistan. *; 2 *Jinnah Postgraduate Medical Centre, Karachi, Pakistan. *; 3 *National Institute of Child Health JPMC, Karachi, Pakistan. *; 4 *Dow University of Health Sciences, Karachi, Pakistan. *; 5 *Ziauddin University Hospital, Karachi, Pakistan. *; 6 *Zainab Punjwani Hospital, Karachi, Pakistan. *; 7 *Baqai Medical University Hospital, Karachi, Pakistan.*; 8 *Care Point Health System New Jersey, USA.*; 9 *Pakistan Health Research Council Research Centre, Karachi, Pakistan. *; 10 *Atomic Energy Medical Center JPMC, Karachi, Pakistan. *

**Keywords:** Cancer, registry, incidence, Karachi, Pakistan

## Abstract

**Objectives:**

To estimate the cancer incidence by age group and gender for the population of Karachi Division by analyzing the Karachi Cancer Registry data of 2017-19.

**Settings:**

The population of Karachi division is 16.1 million according to national census 2017. ‘Karachi Cancer Registry’ which is a part of ‘National Cancer Registry’ is collecting data from eight major hospitals in Karachi since 2017. For outcome measures, cancer counts and the age standardized incidence rates (ASIR) per 100,000 population were computed for age groups (0–14, 15–19 and ≥20 years), in both genders and all cancer site/type.

**Methods:**

The population denominators were based on the population of Karachi division estimated at 16.1 million in the population census, 2017. Counts and age-standardized incidence rates (ASIR) were calculated for each of the three age categories.

**Results:**

From Jan 2017 till Dec 2019 a total of 33,309 malignant cases were recorded in KCR database comprising 17,490 (52.5%) females and 15,819 (47.5%) males. ASIRs in age groups 0-14, 15-19 and ≥ 20 years, among female were 11.5, 2.4 and 223.6 and in males were 17.6, 3.2 and 216.7 respectively. The commonest diagnosis in children, adolescent and adults were (1) among females: children; bone (3.12), leukemia (2.09) brain/CNS (1.26); in adolescents: bone (0.78), brain/CNS (0.27), connective and soft tissue (0.11), in adults: breast cancer (76.07), oral cancer (16.68) and ovary (10.89) respectively, and (2) among males: children; bone (4.56), leukemia (2.79) and brain/CNS (1.88); in adolescent; bone (1.19), brain/CNS (0.31) and leukemia (0.21) and in adults: oral cancer (42.83), liver (16.10) and bone (13.37) respectively.

**Conclusion:**

Oral Cancer, a largely preventable cancer is the leading cancer in Karachi adult males while in female adults Breast Cancer is the leading cancer followed by Oral Cancer. In children and adolescents Bone, Leukemia and Brain/CNS malignancies are most common.

## Introduction

Pakistan is the 5^th^ most populous country in the world with an estimated population of 220 million (Census of Pakistan, 2017). Pakistani population is quite heterogeneous and its four major provinces i.e., Punjab, Sindh, KP and Baluchistan are significantly different and distinct in terms of language, ethnicity, lifestyle and dietary habits, resulting in wide variation and differences in various types of malignancies. Karachi, the port city of Pakistan and capital of Sindh province is the largest city with an estimated population of 16.1 million and rank among ten most populous cities of the world (Census of Pakistan, 2017; Karachi Population, 2020). It is also regarded as ‘Mini Pakistan’ as it has significant representation of all ethnic groups from across Pakistan over and above its native population which include about 45% of migrant population and their subsequent generations who settled in Karachi at the time of partition of sub-continent in 1947 (Census of Pakistan, 2017). This city is like a magnet with huge influx from rest of the country as it is the major industrial and commercial hub of country. Approximately half of its population lives in slums under poor hygienic conditions including water and sanitation issues. Almost all kinds of environmental hazards play their role for various types of ‘Cancers’ in this urban jungle.

Pakistan established its first cancer registry in 1960s at ‘Armed Forces Institute of Pathology (AFIP)’, Rawalpindi. Later from 1970s to 1990s, ‘Pakistan Medical Research Council (PMRC)’ established the ‘National Cancer Registry (NCR)’ (Zaidi et al., 1974; PMRC, 1977). The registry could not sustain due to funding issues. Meanwhile from 1995-2007 a population-based cancer registry was established in Karachi (South) led by Yasmin Bhurgri (Bhurgri, 2004; Bhurgri et al., 2006) and data was shared with IARC and WHO. This registry stopped functioning following her demise almost a decade ago. Meanwhile Lahore, capital of Punjab province and the 2nd largest city of Pakistan established ‘Punjab Cancer Registry’(PKR) (PCR ) which was initiated from the public sector hospital but was later shifted to private sector ‘Shaukat Khanum Memorial Cancer Hospital (SKMCH)’ (PCR, 2005; Badar et al., 2020) which was not only collecting its own data but also from other major hospitals in and around Lahore. This registry is also the source of data to IARC (WHO) and ‘Globocan 2018’ (Bray et al., 2018; Ferlay et al., 2018). In 2015, ‘Ministry of National Health Services Regulations and Coordination’ made ‘PMRC’ now renamed as ‘Pakistan Health Research Council (PHRC)’ the custodian of ‘NCR’.

During all these years, lack of data from Karachi was badly felt and in January 2017; ‘Karachi Cancer Registry (KCR)’ was established as part of NCR with its secretariat at the PHRC, JPMC, Karachi. KCR has collaboration with all major public and private sector hospitals from all over Karachi and is successfully collecting data since 2017 from adults and children. 

## Materials and Methods

*Methodology*

In anticipation of the immense importance of KCR and its functioning on sustainable basis, it was unanimously agreed by all collaborators that there should be a standardized data collection tool which should collect basic data with minimum input. At PHRC, JPMC, there is a trained bio-statistician who has been affiliated with cancer data collection and analysis since 1980’s. He was attached to the KCR to maintain all cancer data received from various Karachi hospitals under strict confidence and resolve that no patient identifiable data shall be released without the approval of the governing body which was constituted having due representation of all participating centers. Data was collected from participating hospitals on either standard paper-based forms (cancer notification form) or as soft copy on Excel sheet while it was agreed that in future, CANREG will be used after proper training of data collection officers of participating hospitals. The hospitals that shared their data included ‘Aga Khan University Hospital’, Jinnah Postgraduate Medical Centre’, ‘Indus Hospital’, ‘National Institute of Child Health’, Dow University of Health Sciences Hospital, Ziauddin University Hospital, Zainab Panjwani Hospital and Baqai Medical University Hospital. Data from some private consultants has been merged with the data from the hospital of their choice so that their data is also represented. Epidemiological data of all malignancies having tissue diagnosis and registered at the Karachi Cancer Registry during 1st January 2017 to 31st December 2019 were analyzed. The data were classified using ICD-10 (International Classification of Diseases-Oncology). As per 2017 census the population of Karachi division was 16.1 million comprising 8.4 million males 7.6 million female. Counts and age-standardized incidence rates (ASIR) were calculated for each of the three age categories (0-14, 15-19 and ≥20 years) The ASIRs were computed using the World Standard of Segi as the standard population, by applying the direct method of age standardization and all the rates presented as per 100,000 population. Data were analyzed using Microsoft Excel and SPSS V.20.

## Results

From 2017-2019 (3-years), data of 33,309 malignant cases were received from eight participating centers/hospitals. There were 17,490 (52.5%) males and 15,819 (47.5%) females. Table 1 and Table 2 shows Age-Standardized incidence rate (ASIR) among females and males by age group (0–14, 15–19 and ≥20 years) of all cancer sites. Table 3 shows ASIR per 100,000 populations in all age groups in comparison with cancer incidence in Lahore district and earlier data of Karachi district.

**Figure 1:**
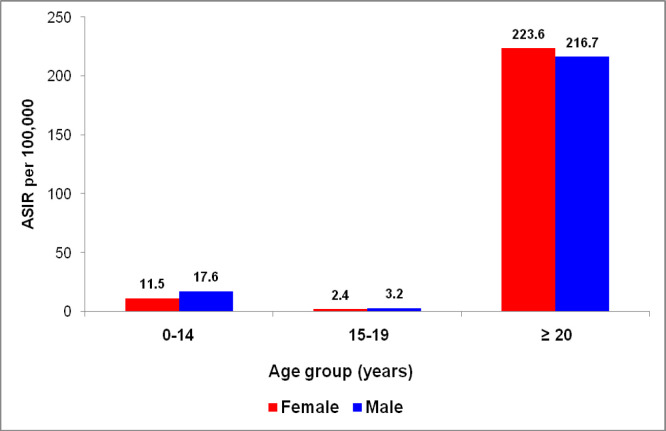
Age Standardized Incidence Rates (ASIR) by Age Group and Gender, in the KCR, Pakistan 2017-2019

**Figure 2:**
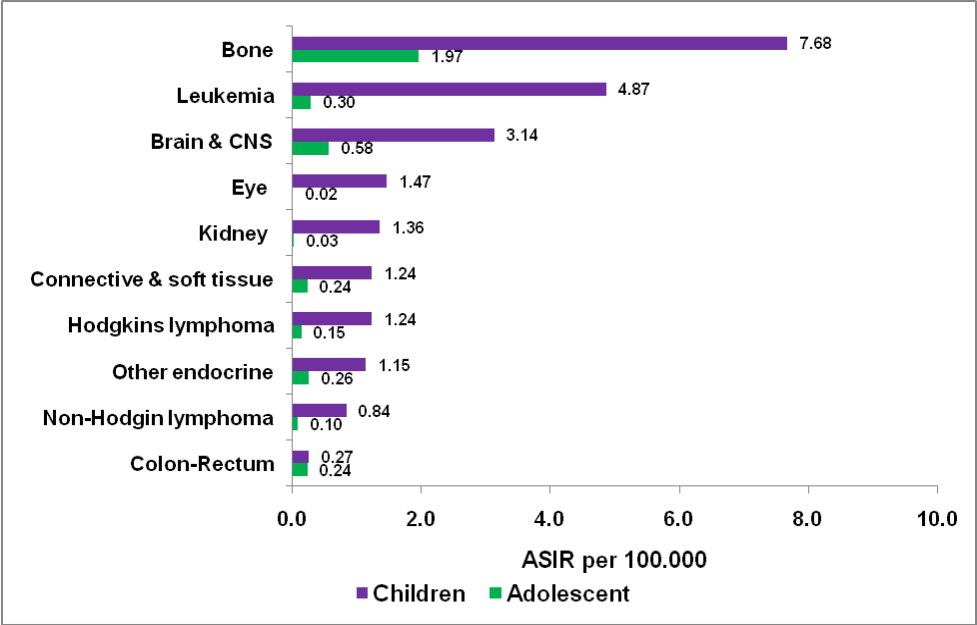
Age Standardized Incidence rates (ASIR) in Children and Adolescent, in the KCR, Pakistan 2017-2019

Age-standardized incidence rates (ASIR) by age group i.e., children, adolescents and adults (0–14, 15–19 and ≥20 years) and gender are shown in Figure 1. Figure 2 shows Age-standardized incidence rates (ASIR) in children and adolescents of top-ten cancers. Children and adolescents of both sexes show Bone, Leukemia and Brain/CNS malignancies as the three topmost malignancies with ASIR of 7.68, 4.87 and 3.14 respectively in children and ASIR of 1.97, 0.30 and 0.58 respectively in adolescents. 

**Figure 3:**
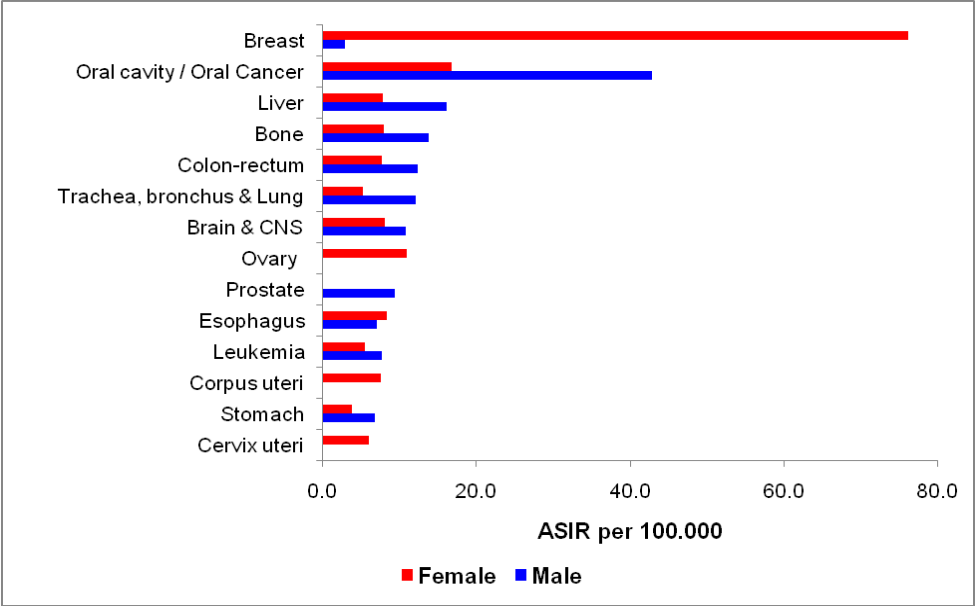
Age Standardized Incidence Rates (ASIR) in Adults in the KCR, Pakistan 2017-2019

Figure 3 shows Age-standardized incidence rates (ASIR) in both females and males’ adults of fourteen topmost cancers. In males, Oral Cancer was the leading malignancy (ASIR 42.83) followed by liver (ASIR 16.10), bone (ASIR 13.37), colorectal (ASIR 12.35), and lung (ASIR 12.01). In female adults Breast Cancer was the leading cancer (ASIR 76.07), followed by Oral Cancer (ASIR 16.68), Ovary (ASIR 10.89), Esophagus (ASIR 8.24), bone (ASIR 7.88) and colorectal (ASIR 7.70).

**Table 1:** Age Standardized Incidence rates (ASIR) among Females, by Age Group, in the KCR, Pakistan 2017-2019

	0-14 years		15-19 years		≥ 20 years		
Site (Females)	Count	ASIR	Count	ASIR	Count	ASIR	ICD-10
Oral cavity / Oral Cancer	7	0.08	7	0.08	1083	16.68	C00-C06
Salivary Gland	5	0.05	0	0	79	1.22	C07-C08
Oropharynx	1	0.01	0	0	54	0.83	C09-C10
Nasopharynx	6	0.06	3	0.03	50	0.77	C11
Hypoharynx	1	0.01	0	0	99	1.52	C12-C14
Esophagus	0	0	1	0.01	535	8.24	C15
Stomach	0	0	2	0.02	249	3.83	C16
Small Intestine	4	0.04	0	0	41	0.63	C17
Colon-rectum	11	0.12	9	0.1	500	7.7	C18-C21
Liver	6	0.06	0	0	503	7.75	C22
Gall bladder & extra hepatic bile duct	0	0	0	0	226	3.48	C23-C24
Pancreas	0	0	2	0.02	153	2.36	C25
Spleen	0	0	0	0	1	0.02	C26
Nose, sinuses, etc.	5	0.05	1	0.01	22	0.34	C30-C31
Larynx	1	0.01	0	0	85	1.31	C32
Trachea, Bronchus & Lung	2	0.02	6	0.07	333	5.13	C33-34
Bone	290	3.12	72	0.78	512	7.88	C42-C43
Skin	8	0.09	4	0.04	207	3.19	C43-C44
Kaposi’s Sarcoma	0	0	0	0	2	0.03	C46
Retroperitonium	5	0.05	0	0	36	0.55	C48
Connective & soft tissue	46	0.49	10	0.11	183	2.82	C47,C49
Breast	0	0	0	0	4940	76.07	C50
Vulva & Vagina	0	0	0	0	61	0.94	C51-C52
Cervix uteri	0	0	0	0	391	6.02	C53
Corpus uteri	0	0	0	0	488	7.51	C54
Uterus	0	0	0	0	118	1.82	C55
Ovary & uterine adnexa	0	0	0	0	707	10.89	C56-C57
Placenta	0	0	0	0	10	0.15	C58
Kidney & other urinary organ	66	0.71	2	0.02	119	1.83	C64-C66
Urinary bladder	1	0.01	0	0	99	1.52	C67
Eye / Orbit	67	0.72	0	0	41	0.63	C69
Brain & CNS	117	1.26	25	0.27	523	8.05	C70-C72
Thyroid gland	1	0.01	4	0.04	227	3.5	C73
Other endocrine	55	0.59	10	0.11	103	1.59	C74-C75
Hodgkin’s lymphoma	27	0.29	7	0.08	49	0.75	C81
Non-Hodgkin lymphoma	19	0.2	3	0.03	137	2.11	C82-85
Multiple myeloma	0	0	0	0	20	0.31	C88,C90
Leukemia	194	2.09	8	0.09	353	5.44	C91-95
Others and unspecified	128	1.31	47	0.46	1184	18.23	
All sites (n)	1073	11.53	223	2.42	14523	223.63	
(%)	6.80%		1.40%		91.80%		

**Table 2:** Age Standardized Incidence Rates (ASIR) among Males, by Age Group, in the KCR, Pakistan 2017-2019

	0-14 years		15-19 years		≥ 20 years		
Site (males)	Count	ASIR	Count	ASIR	Count	ASIR	ICD-10
Oral cavity / Oral Cancer	4	0.04	2	0.02	3024	42.83	C00-C06
Salivary Gland	5	0.05	1	0.01	90	1.27	C07-C08
Oropharynx	1	0.01	0	0	93	1.32	C09-C10
Nasophyraynx	8	0.08	6	0.06	116	1.64	C11
Hypoharynx	1	0.01	0	0	126	1.78	C12-C14
Esophagus	0	0	1	0.01	495	7.01	C15
Stomach	0	0	2	0.02	482	6.83	C16
Small Intestine	7	0.07	0	0	44	0.62	C17
Colon-rectum	16	0.15	14	0.14	872	12.35	C18-C21
Liver	20	0.19	2	0.02	1137	16.1	C22
Gall bladder & extra hepatic bile duct	0	0	0	0	192	2.72	C23-C24
Pancreas	0	0	2	0.02	209	2.96	C25
Spleen	0	0	0	0	2	0.03	C26
Nose, sinuses, etc.	13	0.12	2	0.02	36	0.51	C30-C31
Larynx	3	0.03	0	0	410	5.81	C32
Bronchus & Lung	2	0.02	0	0	848	12.01	C33-34
Thymus, heart, medistrium & pleura	18	0.17	2	0.02	62	0.88	C37-C38
Bone	483	4.56	122	1.19	944	13.37	C42-C43
Skin	9	0.08	6	0.06	342	4.84	C43-C44
Mesothelioma	0	0	0	0	12	0.17	C45
Connective & soft tissue	79	0.75	13	0.13	304	4.31	C47,C49
Retroperitonium	4	0.04	1	0.01	27	0.38	C48
Breast	0	0	0	0	198	2.8	C50
Penis	0	0	0	0	7	0.1	C60
Prostate	0	0	0	0	664	9.4	C61
Testis	0	0	0	0	155	2.2	C62
Other & unspecified male genital organs	0	0	0	0	2	0.03	C63
Kidney & other urinary organ	69	0.65	3	0.03	302	4.28	C64-C66
Urinary bladder	7	0.07	0	0	402	5.69	C67
Eye / Orbit	79	0.75	2	0.02	70	0.99	C69
Brain & Central Nervous System	199	1.88	32	0.31	765	10.83	C70-C72
Thyroid gland	1	0.01	5	0.05	131	1.86	C73
Other endocrine	59	0.56	15	0.15	163	2.31	C74-C75
Hodgkin’s lymphoma	101	0.95	7	0.07	102	1.44	C81
Non-Hodgkin lymphoma	68	0.64	7	0.07	282	3.99	C82-85
Multiple myeloma	0	0	0	0	35	0.5	C88,C90
Leukemia	295	2.79	21	0.21	542	7.68	C91-95
Others and unspecified	310	2.92	62	0.61	1612	22.8	
All sites (n )	1861	17.57	330	3.23	15299	216.66	
(%)	10.60%		1.90%		87.50%		

**Table 3:** ASIR per 100,000 Population, as Reported in Other Studies Conducted in Pakistan

Cancer type/site	KCR 2017-2019 Karachi		PCR 2010-2012 Lahore		Badar et al, 2015 SKMCH&RC, Lahore		Badar et al, 2008 SKMCH&RC, Lahore		KCR 1998-2002 Karachi South District	
	F	M	F	M	F	M	F	M	F	M
0-14 years										
Bone	3.1	4.6	0.5	0.6	0.4	0.4	0.3	0.4	0.7	0.6
Leukemia	2.1	2.8	1.6	2.7	1.5	2	0.5	0.7	1.4	3
Brain & CNS	1.3	1.9	0.5	0.8	1	1	0.3	0.7	1.3	1.5
Eye	0.7	0.8	0.5	0.5	0.4	0.6	0.2	0.3	0.3	0.4
Kidney	0.7	0.7	0.3	0.3	0.1	0.5	0.3	0.3	0.3	0.5
Other endocrine	0.6	0.6	-	-	-	-	-	-	-	-
Connective & soft tissue	0.5	0.8	0.3	0.4	0.2	0.3	0.2	0.4	0.3	0.4
Hodgkin’s lymphoma	0.3	1	0.3	1.1	0.2	0.9	0.2	0.4	0.5	1.4
Non-Hodgkin lymphoma	0.2	0.6	0.3	0.9	0.4	0.7	0.3	0.9	0.8	1.2
Colon-rectum	0.1	0.2	-	-	-	-	-	-	-	-
15-19 years										
Bone	0.8	1.2	1.4	2.4	1	2	-	-	3.3	24
Brain & CNS	0.3	0.3	0.9	1.2	1.7	1.9	-	-	1.6	2.2
Connective & soft tissue	0.1	0.1	0.6	0.9	1	1.3	-	-	0.5	1.8
Other endocrine	0.1	0.2	-	-	-	-	-	-	-	-
Colon-rectum	0.1	0.1	0.5	0.9	-	-	-	-	1.2	1.2
Leukemia	0.1	0.2	0.3	0.9	0.3	0.2	-	-	0.7	0.2
Oral cavity / Oral Cancer	0.1	0	-	-	-	-	-	-	-	-
Hodgkin’s lymphoma	0.1	0.1	0.4	0.1	1.2	1.3	-	-	0.5	0.8
Non-Hodgkin lymphoma	0	0.1	0.5	1.3	0.7	1.3	-	-	2.1	4.2
Skin	0	0.1	0.1	0.3	0	0	-	-	-	0.4
Thyroid gland	0	0.1	-	-	-	-	-	-	-	-
≥ 20 years										
Breast	76.1	2.8	79.2	1.3	-	-	-	-	114.9	1.6
Oral cavity / Oral Cancer	16.7	42.8	6.3	7.6	-	-	-	-	33.7	37.2
Liver	7.8	16.1	4	6.1	-	-	-	-	6.1	8.9
Bone	7.9	13.4	-	-	-	-	-	-	-	-
Colon-rectum	7.7	12.4	6	7.5	-	-	-	-	7.8	10.6
Trachea, bronchus & lung	5.1	12	2	7.7					5.9	41.9
Ovary	10.9	-	7.9	-	-	-	-	-	14.1	-
Brain & CNS	8.1	10.8	3.3	5.8	-	-	-	-	3.5	4.3
Prostate	-	9.4	-	10.7	-	-	-	-	-	16.8
Esophagus	8.2	7	-	-	-	-	-	-	-	-
Leukemia	5.4	7.7	-	-	-	-	-	-	-	-
Stomach	3.8	6.8	-	-	-	-	-	-	-	-
Corpus uteri	7.5	-	6.1						11.1	-
Cervix uteri	6	-								

## Discussion

The KCR data shows ‘Breast Cancer (BC)’ as the most common cancer overall with ‘Oral Cancer (OC)’ as the commonest cancer in males and 2nd commonest in females of Karachi. In children Bone, leukemia and brain/CNS malignancies top the list. Consistent increase in the occurrence of OC in particular in males has been reported earlier also (Zaidi et al., 1974; PMRC, 1977; Bhurgri, 2004; Bhurgri, 2005; Bhurgri et al., 2006) with majority (95%) of them having a chronic history of chewing or/and smoking (Bhurgri, 2005; Pervez and Abro, 2017). The data from ‘Punjab Cancer Registry (PCR)’, available as 2018 Globocan Cancer Data estimates for Pakistan also show similar prevalence for oral and other cancers (Bray et al., 2018; Ferlay et al., 2018). Similarly, the monograph from ‘Pakistan Atomic Energy Commission (PAEC)’ for 2015-17 also shows a very similar pattern (PAEC, 2019). Rampant chewing habits including Areca nut (Chaalia), Tobacoo (Tambakoo) and Betel leaf (Paan) is implicated for being carcinogenic. Oral Submucosal Fibrosis (OSF), a precancerous condition for OC largely restricted to Indian sub-continent is also an established condition to have a direct correlation with areca nut chewing. More recently chewing of some readymade products termed ‘Gutka’ and ‘Mainpuri’ has largely replaced above-mentioned traditional chewing habits in Pakistan. Gutka and Mainpuri is a highly addictive, ready-made psychoactive stuff which is widely used by all genders and ages including young children and adolescents. It contains crushed areca nut, tobacco in various chewable forms, lime, catechu (kattha), wax and several flavoring agents (Abro and Pervez, 2017). Unlike traditional Paan where one takes one Paan at a time and can adjust the quantity of the ingredients according to the choice and where the chewed quid is mostly spitted out, in Gutka and Mainpuri, most stuff comes as mixed grounded powder in bulk which one can take as often as one may like with convenient storage. The powder is kept in the mouth for hours and then the quid is either spitted out or gulped depending on the choice. Hence, it is the prolonged exposure of the oral mucosa to the carcinogenic contents of the quid that predisposes to precancerous lesions and eventually OC. Naswar is another smokeless tobacco that is taken orally mostly by ethnic Pathans and Balochs residing in Karachi from KPK and Baluchistan Provinces. In Naswar a pinch of wet product is placed between gums and cheek. It is prepared by grounding green tobacco leaves that are intermixed with lime and other stuff (Abro and Pervez, 2017., Pervez and Abro, 2017). Comparative data of KCR 2017-19 vs. 1998-2002 (Table 3) show that although OC is still the 2nd most common cancer in females of Karachi, there is a decline in ASIR in females of Karachi compared to 1998-2002. This is assumed that this is largely due to explosive increase in chewing among males of Karachi who are showing more and more inclination to abandon smoking and acquire chewing. This may partially be due to social pressure as smoking is being less and less socially accepted and little space is left for the smokers to freely smoke. In contrast chewing is still very much accepted in the society at large as no smoke is released, hence no such danger for those sitting nearby. In addition cost may be another factor as with each passing year taxes are increased on cigarettes. In contrast smoking among females of Karachi historically has been very low, hence no such shift is seen. In northern Pakistan although chewing habits are relatively much less prevalent, OC still ranks among topmost cancers. It is therefore fair to conclude that one of the major cancers of Pakistan i.e. OC is largely preventable. 

On the same note two other common cancers in Karachi i.e., Lung Cancer and Liver cancer are also largely preventable cancers. Almost 80% patients with lung cancer have a history of ‘Smoking’ therefore refraining from smoking is the largest prevention strategy for lung cancer. Most primary liver cancers (Hepatocellular carcinoma, HCC) in Pakistan are due to ‘Chronic Hepatitis B (Qureshi et al., 1990; Qureshi et al., 2010) as well as Hepatitis C (Capileno et al., 2017; Pervez., 2011). Liver cancer is a highly aggressive cancer with a very high mortality in developing countries like ours. Ironically again Hepatitis B is a preventable disease as vaccine is available for a long time for mass immunization of children and adults. Safe blood transfusion may do the rest for both hepatitis B and C. The association of ‘aspergillus’ a fungus which secretes ‘aflatoxin’ that is toxic to liver and is carcinogenic is also studied in Karachi (Nizami et al, 1986). It was observed that the spices and other food commodities sold in open market under hot and humid climate of Karachi were contaminated with this fungus. So, this etiology is also preventable. 

BC which is the topmost cancer in women like many other non-preventable cancers may arise out of nowhere, this is also termed as ‘biological bad luck’ and largely falls in the category of non-preventable cancer (Pervez, 2019). Thought it is largely non-preventable, its occurrence can be lowered by healthy lifestyle and controlling of risk factors like obesity. Heredity also could not explain such high occurrence of BC based on few studies done on local patients for *BRCA1* mutations (Moatter et al., 2011; Rashid et al., 2017). On the face of such a high burden of BC, only option is early detection of breast cancer by a massive screening program catering the women at all levels of health care. 

Lung Cancer has shown a consistent downward trend since 1995-1997, when it was the leading cancer in males, by 1998-2002 it gave way to OC as the leading cancer. Since then it has shown a downward trend and by 2017-19, its frequency is significantly dwarfed compared to OC. It is assumed that the major factor responsible for this change is the paradigm shift in lifestyle in southern Pakistan (Sindh) including its capital Karachi where ‘Smoking’ gave way to ‘Chewing’ in a big way (Majeed et al., 2019).

‘Colorectal Cancer’ is also among top-most cancers in both males and females. This cancer is thought to be historically more common in western population whose diet is rich in fat and red meat and low in fiber (Yaha et al., 2013). This is alarming and points an adversity of globalization with increasing consumption of fast and junk food including smoked red meat (Jahan et al., 2014). 

Comparison of cancer incidence in Karachi, Pakistan, to the figures from India show quite similar pattern, i.e., Lip and oral cavity cancer are the most frequent cancer in males i.e., 17.32 % KCR vs. 16.1%.) and BC as most common cancer in females i.e.,31.27% KCR vs. 27.7%) (Bhurgri et al., 2003., Bray et al., 2018; Ferlay et al., 2018). However, in contrast to Lip and oral cavity cancer in females of Karachi which is ranked 2^nd^ most common cancer (6.93%), in India it is ranked 4^th^ (4.8%) with cervix uteri cancer as the 2^nd^ most common cancer i.e., (16.5% vs. 2.48% in KCR). Ovarian cancer ranks 3^rd^ in Pakistan and India, (4.61% in KCR vs 6.2%) (Bray et al., 2018; Ferlay et al., 2018). These similarities could be due to the fact that the most dominant population of Karachi represent migrants (also called Mohajirs or Urdu-Speaking) from all parts of India at the time of partition and their pedigree, hence certain life style factors like chewing habits are extremely prevalent on both sides of the border. Bangladesh which is a major producer of ‘betel leaf’ also has OC as the 2nd most common cancer in both genders while esophageal cancer is ranked as the leading cancer in Bangladeshi males (Bray et al., 2018; Ferlay et al., 2018). In one province of Pakistan (Baluchistan, which borders both Afghanistan and Iran) however Esophageal Cancer (EC) is the most common cancer in males and the 2^nd^ most common cancer in females. This was also shown in the Afghani males and females who regularly come to PAEC for cancer treatment (Mummudi et al., 2019). This observation was also reported earlier when incidence of EC in Quetta was compared with Karachi south and it was concluded that the incidence of EC in Quetta was comparable to that of high-incidence regions like Afghanistan and Iran, (Bhurgri et al., 2003., PAEC, 2019).

Cervical cancer is relatively uncommon in Karachi and Pakistan as 98% of Pakistani population is Muslim and supposedly less promiscus (Bhurgri et al., 2007). Ironically ‘Leukemia’ incidence in adults is also on the rise and is ranked no.5 in both males and females of Karachi and ranks 7th in India (Bray et al., 2018; Ferlay et al., 2018).

Leukemias and lymphomas have been reported very frequently in adults from Northern Pakistan. The KPK province of Pakistan shows a very high prevalence of hematolymphoid malignancies (Leukemia and Lymphoma) (PAEC, 2019). 

As far as children and adolescent cancer prevalence is concerned as these are largely non-preventable cancers which arise from fatal genetic mutations and chromosomal translocations, patterns similar to rest of the world are seen i.e., Bone malignancies, Hematopoietic malignancies like ‘Leukemia’ and ‘Lymphoma’ and Brain/CNS ‘ (Steliarova-Foucher et al., 2018).

In Summary for any ‘Cancer Control Program (CCP)’ foremost requirement is availability of reliable cancer incidence data from which major risk factors in a population may be deduced. Therefore ‘Cancer Registries (CRs)’ are the backbone of any meaningful CCP for all countries. Cancer treating facilities in Pakistan are mostly attached to the tertiary care hospitals across nation where hospitals are choked with patients diagnosed with various types of cancers, mostly at the advanced stage where palliation and the relief of pain is the only option while sufferings are huge and cost to the patient and health care system are largely not met. (Nishter et al., 2004) In a populous country like Pakistan with marked ethnic and lifestyle variability, regional cancer registries like KCR are very important for cancer risk assessment and its categorization into modifiable and non-modifiable factors.
